# Hexaaqua­cobalt(II) tetra­aqua­bis(2-amino­pyrazine-κ*N* ^4^)cobalt(II) disulfate dihydrate

**DOI:** 10.1107/S1600536809045310

**Published:** 2009-11-04

**Authors:** Wei Kang, Li-Hua Huo, Shan Gao, Seik Weng Ng

**Affiliations:** a College of Chemistry and Materials Science, Heilongjiang University, Harbin 150080, People’s Republic of China; b Department of Chemistry, University of Malaya, 50603 Kuala Lumpur, Malaysia

## Abstract

The reaction of cobalt(II) sulfate and 2-amino­pyrazine affords the title salt, [Co(H_2_O)_6_][Co(C_4_H_5_N_3_)_2_(H_2_O)_4_](SO_4_)_2_·2H_2_O. The metal atoms in the tetra­aqua-coordinated and hexa­aqua-coordinated complex cations lie on centers of inversion in slightly distorted octa­hedral geometries. The cations, anions and solvent water mol­ecules are linked by O—H⋯O, O—H⋯N and N—H⋯O hydrogen bonds into a three-dimensional network.

## Related literature

The reaction of cobalt(II) chloride and 3-amino­pyrazine yields tetra­kis(3-amino­pyrazine)dichloridocobalt(II); see: Csöregh *et al.* (2000 ▶); Kang *et al.* (2009 ▶).
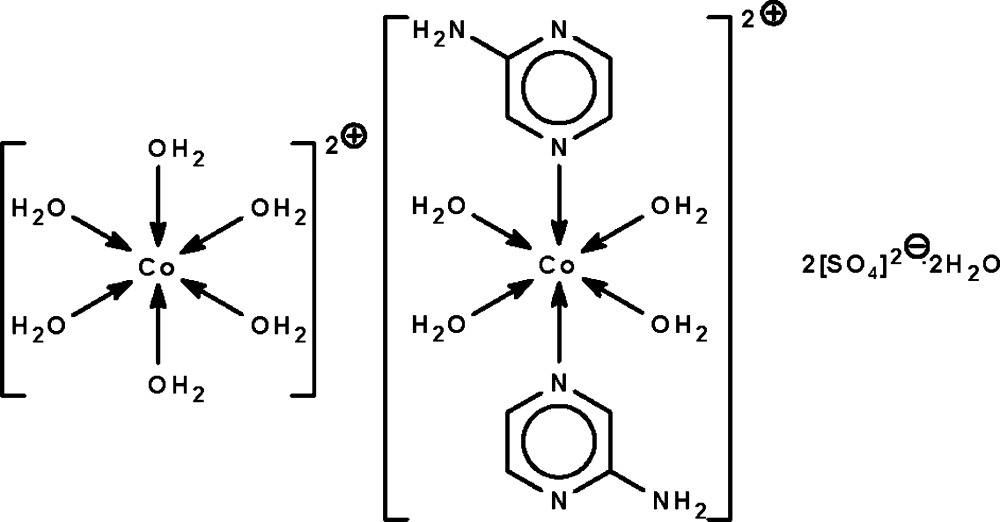


## Experimental

### 

#### Crystal data

[Co(H_2_O)_6_][Co(C_4_H_5_N_3_)_2_(H_2_O)_4_](SO_4_)_2_·2H_2_O
*M* *_r_* = 716.40
Triclinic, 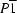

*a* = 6.5722 (3) Å
*b* = 8.3264 (4) Å
*c* = 13.2337 (7) Å
α = 75.732 (2)°
β = 78.571 (1)°
γ = 78.795 (1)°
*V* = 679.81 (6) Å^3^
*Z* = 1
Mo *K*α radiation
μ = 1.47 mm^−1^
*T* = 293 K
0.30 × 0.20 × 0.20 mm

#### Data collection

Rigaku R-AXIS RAPID IP diffractometer
Absorption correction: multi-scan (*ABSCOR*; Higashi, 1995 ▶) *T*_min_ = 0.668, *T*_max_ = 0.758
6692 measured reflections
3071 independent reflections
2762 reflections with *I* > 2σ(*I*)
*R*_int_ = 0.027

#### Refinement

*R*[*F* ^2^ > 2σ(*F* ^2^)] = 0.031
*wR*(*F* ^2^) = 0.085
*S* = 1.05
3071 reflections
231 parameters
14 restraints
H atoms treated by a mixture of independent and constrained refinement
Δρ_max_ = 0.47 e Å^−3^
Δρ_min_ = −0.33 e Å^−3^

### 

Data collection: *RAPID-AUTO* (Rigaku, 1998 ▶); cell refinement: *RAPID-AUTO*; data reduction: *CrystalClear* (Rigaku/MSC, 2002 ▶); program(s) used to solve structure: *SHELXS97* (Sheldrick, 2008 ▶); program(s) used to refine structure: *SHELXL97* (Sheldrick, 2008 ▶); molecular graphics: *X-SEED* (Barbour, 2001 ▶); software used to prepare material for publication: *publCIF* (Westrip, 2009 ▶).

## Supplementary Material

Crystal structure: contains datablocks global, I. DOI: 10.1107/S1600536809045310/xu2657sup1.cif

Structure factors: contains datablocks I. DOI: 10.1107/S1600536809045310/xu2657Isup2.hkl

Additional supplementary materials:  crystallographic information; 3D view; checkCIF report

## Figures and Tables

**Table 1:** Hydrogen-bond geometry (Å, °)

*D*—H⋯*A*	*D*—H	H⋯*A*	*D*⋯*A*	*D*—H⋯*A*
O1w—H1w1⋯O1	0.84 (1)	1.93 (1)	2.755 (2)	168 (3)
O1w—H1w2⋯N2^i^	0.85 (1)	1.95 (1)	2.795 (2)	175 (3)
O2w—H2w1⋯O3	0.84 (1)	1.94 (1)	2.769 (2)	170 (2)
O2w—H2w2⋯O1^ii^	0.84 (1)	1.93 (1)	2.765 (2)	170 (3)
O3w—H3w1⋯O2	0.85 (1)	1.91 (1)	2.743 (2)	169 (3)
O3w—H3w2⋯O6w	0.85 (1)	1.89 (1)	2.730 (2)	170 (3)
O4w—H4w1⋯O6w^iii^	0.85 (1)	1.95 (1)	2.781 (2)	168 (3)
O4w—H4w2⋯O2^iii^	0.85 (1)	1.91 (1)	2.745 (2)	167 (2)
O5w—H5w1⋯O3^iv^	0.85 (1)	1.98 (1)	2.816 (2)	170 (3)
O5w—H5w2⋯O4^v^	0.84 (1)	1.90 (1)	2.737 (2)	174 (3)
O6w—H6w1⋯O3^i^	0.85 (1)	1.94 (1)	2.782 (2)	171 (4)
O6w—H6w2⋯O4^iv^	0.85 (1)	1.89 (1)	2.711 (2)	164 (3)
N3—H3n2⋯O1^vi^	0.85 (1)	2.20 (1)	3.036 (2)	168 (3)

Symmetry codes: (i) 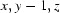; (ii) 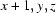; (iii) 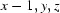; (iv) 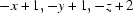; (v) 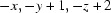; (vi) 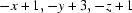.

**Fig. 1.**
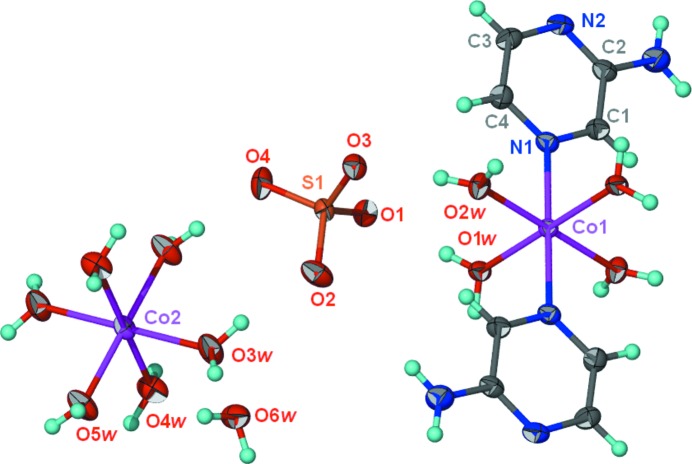
Thermal ellipsoid plot (Barbour, 2001) of [Co(H2O)6] [Co(H2O)4(C4H5N3)2] 2[SO4].2H2O at the 50% probability level; hydrogen atoms are drawn as spheres of arbitrary radius.

## supplementary crystallographic information

### Experimental

To an aqueous solution of 3-aminopyrazine (0.19 g, 2 mmol) was added cobalt(II) sulfate heptahydrate (0.56 g, 2 mmol). Red crystals of the salt separated from the solution after a few days. CH&N elemental analysis. Calc. for C_8_H_34_N_6_O_20_S_2_Co_2_: C 13.41, H 4.78, N 11.73%; found: C 13.39, H 4.72, N 11.76%.

### Refinement

Carbon-bound H-atoms were placed in calculated positions (C—H 0.93 Å) and were included in the refinement in the riding model approximation, with *U*(H) set to 1.2*U*(C). The amino and water H-atoms were located in a difference Fourier map, and were refined with a distance restraint of N–H = O–H = 0.85±0.01 Å; their temperature factors were refined.

### Crystal data

**Table:**

[Co(H_2_O)_6_][Co(C_4_H_5_N_3_)_2_(H_2_O)_4_](SO_4_)_2_·2H_2_O	*Z* = 1
*M**_r_* = 716.40	*F*(000) = 370
Triclinic, *P*1	*D*_x_ = 1.750 Mg m^−^^3^
Hall symbol: -P 1	Mo *K*α radiation, λ = 0.71073 Å
*a* = 6.5722 (3) Å	Cell parameters from 6326 reflections
*b* = 8.3264 (4) Å	θ = 3.2–27.5°
*c* = 13.2337 (7) Å	µ = 1.47 mm^−^^1^
α = 75.732 (2)°	*T* = 293 K
β = 78.571 (1)°	Prism, red
γ = 78.795 (1)°	0.30 × 0.20 × 0.20 mm
*V* = 679.81 (6) Å^3^	

### Data collection

**Table:**

Rigaku RAXIS-RAPID IP diffractometer	3071 independent reflections
Radiation source: fine-focus sealed tube	2762 reflections with *I* > 2σ(*I*)
graphite	*R*_int_ = 0.027
ω scans	θ_max_ = 27.4°, θ_min_ = 3.2°
Absorption correction: multi-scan (*ABSCOR*; Higashi, 1995)	*h* = −7→8
*T*_min_ = 0.668, *T*_max_ = 0.758	*k* = −10→10
6692 measured reflections	*l* = −17→17

### Refinement

**Table:**

Refinement on *F*^2^	Primary atom site location: structure-invariant direct methods
Least-squares matrix: full	Secondary atom site location: difference Fourier map
*R*[*F*^2^ > 2σ(*F*^2^)] = 0.031	Hydrogen site location: inferred from neighbouring sites
*wR*(*F*^2^) = 0.085	H atoms treated by a mixture of independent and constrained refinement
*S* = 1.05	*w* = 1/[σ^2^(*F*_o_^2^) + (0.0511*P*)^2^ + 0.1887*P*] where *P* = (*F*_o_^2^ + 2*F*_c_^2^)/3
3071 reflections	(Δ/σ)_max_ = 0.001
231 parameters	Δρ_max_ = 0.47 e Å^−^^3^
14 restraints	Δρ_min_ = −0.33 e Å^−^^3^

### Special details

**Table:**

Geometry. All e.s.d.'s (except the e.s.d. in the dihedral angle between two l.s. planes) are estimated using the full covariance matrix. The cell e.s.d.'s are taken into account individually in the estimation of e.s.d.'s in distances, angles and torsion angles; correlations between e.s.d.'s in cell parameters are only used when they are defined by crystal symmetry. An approximate (isotropic) treatment of cell e.s.d.'s is used for estimating e.s.d.'s involving l.s. planes.

### Fractional atomic coordinates and isotropic or equivalent isotropic displacement parameters (Å^2^)

**Table:**

	*x*	*y*	*z*	*U*_iso_*/*U*_eq_	
Co1	1.0000	1.0000	0.5000	0.02058 (10)	
Co2	0.0000	0.5000	1.0000	0.02713 (11)	
S1	0.48109 (7)	0.88842 (5)	0.80066 (3)	0.02386 (12)	
O1	0.4133 (2)	0.98162 (18)	0.70001 (10)	0.0309 (3)	
O2	0.5369 (3)	0.70948 (18)	0.79992 (14)	0.0439 (4)	
O3	0.6685 (2)	0.95119 (18)	0.81446 (11)	0.0317 (3)	
O4	0.3094 (2)	0.9161 (2)	0.88747 (12)	0.0410 (4)	
O1W	0.7178 (2)	0.91396 (17)	0.53377 (11)	0.0295 (3)	
O2W	1.0213 (2)	0.96509 (18)	0.66021 (11)	0.0305 (3)	
O3W	0.2836 (2)	0.4730 (2)	0.90039 (14)	0.0435 (4)	
O4W	−0.1433 (3)	0.4747 (2)	0.87641 (13)	0.0399 (4)	
O5W	0.0399 (2)	0.23895 (19)	1.05544 (14)	0.0421 (4)	
O6W	0.6169 (3)	0.2195 (2)	0.91230 (13)	0.0397 (3)	
N1	0.8413 (2)	1.25974 (19)	0.49825 (13)	0.0257 (3)	
N2	0.7121 (3)	1.6016 (2)	0.49242 (14)	0.0302 (3)	
N3	0.7876 (3)	1.6554 (2)	0.30956 (16)	0.0423 (4)	
C1	0.8491 (3)	1.3722 (2)	0.40789 (15)	0.0289 (4)	
H1	0.9012	1.3361	0.3452	0.035*	
C2	0.7812 (3)	1.5450 (2)	0.40335 (16)	0.0283 (4)	
C3	0.7021 (3)	1.4852 (3)	0.58309 (16)	0.0317 (4)	
H3	0.6516	1.5207	0.6461	0.038*	
C4	0.7624 (3)	1.3170 (2)	0.58746 (15)	0.0306 (4)	
H4	0.7489	1.2415	0.6523	0.037*	
H1W1	0.615 (3)	0.941 (3)	0.5778 (16)	0.039 (7)*	
H1W2	0.708 (4)	0.822 (2)	0.521 (2)	0.043 (7)*	
H2W1	0.924 (3)	0.961 (3)	0.7118 (14)	0.038 (7)*	
H2W2	1.136 (3)	0.983 (4)	0.671 (2)	0.058 (9)*	
H3W1	0.348 (5)	0.556 (3)	0.872 (3)	0.072 (10)*	
H3W2	0.376 (3)	0.387 (2)	0.906 (2)	0.050 (8)*	
H4W1	−0.199 (5)	0.387 (3)	0.886 (3)	0.068 (10)*	
H4W2	−0.234 (3)	0.558 (2)	0.856 (2)	0.043 (7)*	
H5W1	0.135 (3)	0.176 (3)	1.0881 (19)	0.046 (8)*	
H5W2	−0.062 (4)	0.185 (4)	1.076 (2)	0.066 (9)*	
H6W1	0.637 (6)	0.145 (4)	0.876 (3)	0.094 (13)*	
H6W2	0.625 (5)	0.164 (4)	0.9747 (12)	0.065 (10)*	
H3N1	0.830 (5)	1.618 (4)	0.2538 (15)	0.062 (9)*	
H3N2	0.751 (5)	1.7602 (15)	0.307 (3)	0.064 (9)*	

### Atomic displacement parameters (Å^2^)

**Table:**

	*U*^11^	*U*^22^	*U*^33^	*U*^12^	*U*^13^	*U*^23^
Co1	0.02009 (17)	0.01865 (17)	0.02239 (18)	−0.00235 (12)	−0.00307 (13)	−0.00400 (12)
Co2	0.02194 (19)	0.02479 (19)	0.0323 (2)	−0.00440 (14)	−0.00566 (14)	0.00002 (14)
S1	0.0220 (2)	0.0245 (2)	0.0240 (2)	−0.00519 (17)	−0.00492 (16)	−0.00072 (16)
O1	0.0285 (7)	0.0353 (7)	0.0270 (7)	−0.0006 (6)	−0.0095 (5)	−0.0022 (5)
O2	0.0429 (9)	0.0237 (7)	0.0623 (10)	−0.0039 (6)	−0.0138 (8)	−0.0005 (7)
O3	0.0241 (6)	0.0394 (8)	0.0336 (7)	−0.0094 (6)	−0.0057 (5)	−0.0074 (6)
O4	0.0292 (7)	0.0610 (10)	0.0311 (7)	−0.0127 (7)	0.0028 (6)	−0.0080 (7)
O1W	0.0249 (7)	0.0264 (7)	0.0383 (8)	−0.0067 (5)	0.0030 (6)	−0.0134 (6)
O2W	0.0266 (7)	0.0413 (8)	0.0242 (7)	−0.0062 (6)	−0.0044 (5)	−0.0069 (6)
O3W	0.0280 (8)	0.0322 (8)	0.0594 (10)	−0.0041 (6)	0.0049 (7)	0.0007 (7)
O4W	0.0383 (8)	0.0344 (8)	0.0493 (9)	−0.0060 (7)	−0.0199 (7)	−0.0029 (7)
O5W	0.0314 (8)	0.0290 (8)	0.0618 (10)	−0.0075 (6)	−0.0170 (7)	0.0077 (7)
O6W	0.0501 (9)	0.0321 (8)	0.0387 (9)	−0.0050 (7)	−0.0107 (7)	−0.0089 (7)
N1	0.0231 (7)	0.0206 (7)	0.0326 (8)	−0.0019 (6)	−0.0050 (6)	−0.0053 (6)
N2	0.0271 (8)	0.0234 (8)	0.0425 (9)	−0.0026 (6)	−0.0079 (7)	−0.0104 (7)
N3	0.0564 (12)	0.0247 (9)	0.0399 (11)	0.0015 (8)	−0.0053 (9)	−0.0040 (8)
C1	0.0305 (9)	0.0241 (9)	0.0318 (10)	−0.0029 (7)	−0.0039 (8)	−0.0072 (7)
C2	0.0244 (9)	0.0224 (9)	0.0378 (10)	−0.0034 (7)	−0.0059 (7)	−0.0052 (7)
C3	0.0290 (9)	0.0318 (10)	0.0361 (10)	0.0005 (8)	−0.0061 (8)	−0.0138 (8)
C4	0.0305 (10)	0.0296 (10)	0.0294 (9)	−0.0018 (8)	−0.0040 (8)	−0.0052 (7)

### Geometric parameters (Å, °)

**Table:**

Co1—O1W	2.0451 (13)	O3W—H3W1	0.846 (10)
Co1—O1W^i^	2.0451 (13)	O3W—H3W2	0.846 (10)
Co1—O2W^i^	2.0970 (13)	O4W—H4W1	0.847 (10)
Co1—O2W	2.0970 (13)	O4W—H4W2	0.850 (10)
Co1—N1^i^	2.2076 (15)	O5W—H5W1	0.849 (10)
Co1—N1	2.2076 (15)	O5W—H5W2	0.840 (10)
Co2—O3W	2.0670 (16)	O6W—H6W1	0.849 (10)
Co2—O3W^ii^	2.0670 (16)	O6W—H6W2	0.847 (10)
Co2—O5W	2.0977 (15)	N1—C1	1.324 (2)
Co2—O5W^ii^	2.0977 (15)	N1—C4	1.352 (3)
Co2—O4W	2.1128 (16)	N2—C2	1.340 (3)
Co2—O4W^ii^	2.1128 (16)	N2—C3	1.343 (3)
S1—O2	1.4656 (16)	N3—C2	1.349 (3)
S1—O4	1.4703 (15)	N3—H3N1	0.849 (10)
S1—O1	1.4717 (13)	N3—H3N2	0.852 (10)
S1—O3	1.4866 (14)	C1—C2	1.411 (3)
O1W—H1W1	0.836 (10)	C1—H1	0.9300
O1W—H1W2	0.846 (10)	C3—C4	1.370 (3)
O2W—H2W1	0.838 (10)	C3—H3	0.9300
O2W—H2W2	0.843 (10)	C4—H4	0.9300
			
O1W—Co1—O1W^i^	180.0	Co1—O1W—H1W1	126.4 (17)
O1W—Co1—O2W^i^	87.43 (6)	Co1—O1W—H1W2	120.7 (18)
O1W^i^—Co1—O2W^i^	92.57 (6)	H1W1—O1W—H1W2	110 (2)
O1W—Co1—O2W	92.57 (6)	Co1—O2W—H2W1	128.2 (18)
O1W^i^—Co1—O2W	87.43 (6)	Co1—O2W—H2W2	114 (2)
O2W^i^—Co1—O2W	180.000 (1)	H2W1—O2W—H2W2	116 (3)
O1W—Co1—N1^i^	88.71 (6)	Co2—O3W—H3W1	121 (2)
O1W^i^—Co1—N1^i^	91.29 (6)	Co2—O3W—H3W2	125.2 (19)
O2W^i^—Co1—N1^i^	90.16 (6)	H3W1—O3W—H3W2	107 (3)
O2W—Co1—N1^i^	89.84 (6)	Co2—O4W—H4W1	117 (2)
O1W—Co1—N1	91.29 (6)	Co2—O4W—H4W2	114.2 (18)
O1W^i^—Co1—N1	88.71 (6)	H4W1—O4W—H4W2	107 (3)
O2W^i^—Co1—N1	89.84 (6)	Co2—O5W—H5W1	128.5 (19)
O2W—Co1—N1	90.16 (6)	Co2—O5W—H5W2	122 (2)
N1^i^—Co1—N1	180.000 (1)	H5W1—O5W—H5W2	103 (3)
O3W—Co2—O3W^ii^	180.0	H6W1—O6W—H6W2	104 (3)
O3W—Co2—O5W	88.95 (7)	C1—N1—C4	117.08 (16)
O3W^ii^—Co2—O5W	91.05 (7)	C1—N1—Co1	119.42 (13)
O3W—Co2—O5W^ii^	91.05 (7)	C4—N1—Co1	122.75 (13)
O3W^ii^—Co2—O5W^ii^	88.95 (7)	C2—N2—C3	116.49 (17)
O5W—Co2—O5W^ii^	180.0	C2—N3—H3N1	118 (2)
O3W—Co2—O4W	87.20 (7)	C2—N3—H3N2	121 (2)
O3W^ii^—Co2—O4W	92.80 (7)	H3N1—N3—H3N2	121 (3)
O5W—Co2—O4W	89.82 (7)	N1—C1—C2	122.25 (18)
O5W^ii^—Co2—O4W	90.18 (7)	N1—C1—H1	118.9
O3W—Co2—O4W^ii^	92.80 (7)	C2—C1—H1	118.9
O3W^ii^—Co2—O4W^ii^	87.20 (7)	N2—C2—N3	119.21 (18)
O5W—Co2—O4W^ii^	90.18 (7)	N2—C2—C1	120.28 (18)
O5W^ii^—Co2—O4W^ii^	89.82 (7)	N3—C2—C1	120.50 (19)
O4W—Co2—O4W^ii^	180.0	N2—C3—C4	123.26 (19)
O2—S1—O4	110.72 (10)	N2—C3—H3	118.4
O2—S1—O1	109.81 (9)	C4—C3—H3	118.4
O4—S1—O1	108.83 (9)	N1—C4—C3	120.53 (18)
O2—S1—O3	108.89 (9)	N1—C4—H4	119.7
O4—S1—O3	109.26 (9)	C3—C4—H4	119.7
O1—S1—O3	109.31 (8)		

Symmetry codes: (i) −*x*+2, −*y*+2, −*z*+1; (ii) −*x*, −*y*+1, −*z*+2.

### Hydrogen-bond geometry (Å, °)

**Table:**

*D*—H···*A*	*D*—H	H···*A*	*D*···*A*	*D*—H···*A*
O1w—H1w1···O1	0.84 (1)	1.93 (1)	2.755 (2)	168 (3)
O1w—H1w2···N2^iii^	0.85 (1)	1.95 (1)	2.795 (2)	175 (3)
O2w—H2w1···O3	0.84 (1)	1.94 (1)	2.769 (2)	170 (2)
O2w—H2w2···O1^iv^	0.84 (1)	1.93 (1)	2.765 (2)	170 (3)
O3w—H3w1···O2	0.85 (1)	1.91 (1)	2.743 (2)	169 (3)
O3w—H3w2···O6w	0.85 (1)	1.89 (1)	2.730 (2)	170 (3)
O4w—H4w1···O6w^v^	0.85 (1)	1.95 (1)	2.781 (2)	168 (3)
O4w—H4w2···O2^v^	0.85 (1)	1.91 (1)	2.745 (2)	167 (2)
O5w—H5w1···O3^vi^	0.85 (1)	1.98 (1)	2.816 (2)	170 (3)
O5w—H5w2···O4^ii^	0.84 (1)	1.90 (1)	2.737 (2)	174 (3)
O6w—H6w1···O3^iii^	0.85 (1)	1.94 (1)	2.782 (2)	171 (4)
O6w—H6w2···O4^vi^	0.85 (1)	1.89 (1)	2.711 (2)	164 (3)
N3—H3n2···O1^vii^	0.85 (1)	2.20 (1)	3.036 (2)	168 (3)

Symmetry codes: (iii) *x*, *y*−1, *z*; (iv) *x*+1, *y*, *z*; (v) *x*−1, *y*, *z*; (vi) −*x*+1, −*y*+1, −*z*+2; (ii) −*x*, −*y*+1, −*z*+2; (vii) −*x*+1, −*y*+3, −*z*+1.
